# Remarks on Ether, and Chloroform

**Published:** 1848-05

**Authors:** Caleb Green


ART. II.—*Remarks on Ether, and Chloroform.* By Caleb Green, M. D.

Dr. Flint, Homer, March 14th, 1848.

*Dear Sir*: — I have made a few observations on the effects of sulph. ether and chloroform, which I will state briefly; and if you should judge a knowledge of them would be of any value to the profession, you are at liberty to publish them. I have seen the sulph. ether administered in a few cases by a dentist, and have in one case observed the induction of total insensibility to pain, but not unconsciousness. In the others, there was neither unconsciousness nor insensibility—the patients suffering nearly as much from pain as when not under the influence of ether, although they were decidedly "tipsy." The most obvious effects which I have noticed are, laborious breathing, dilated pupil, pale or flushed face, prostration of strength, at first increased, and afterwards diminished force and frequency of the pulse, and mental aberration. In some cases these effects were followed by headache, but in others the previously existing headache was relieved. I state the cases only that I have witnessed, and my observation has been limited.

---

I have made two or three experiments on the lower animals, with ether. The first was on a cat, six or eight months old. We placed a sponge saturated with ether in a glass jar having a hole in the bottom. This was placed to the nose, and she inhaled it readily. In about half a minute total insensibility was induced, with slow, laborious breathing, greatly dilated pupil, and frothing at the mouth. She recovered in less than half a minute. Two days afterwards we brought her to the table again, and induced total insensibility in less than a minute. I immediately laid open the abdomen and chest, during which the animal gave not the slightest indication of pain. The lungs collapsed, the heart continued feebly in action about five minutes, and the peristaltic action of the intestines continued about half an hour. The brain and venous trunks were fully engorged—the lungs free from congestion. This, by the way, is a very humane and convenient method of displaying the thoracic and abdominal viscera during life. In this instance the lacteals were beautifully exhibited, being full of chyle.

The last experiment was on an old cat. Insensibility was not as readily induced as in the former instance. In about five minutes insensibility was induced, with slow *stertorous* breathing, like apoplexy, dilated pupils, &c. I laid open the chest and abdomen. The right auricle and ventricle were fully distended with black blood. I was unable to excite contraction of the heart, with the exception of three or four feeble contractions of the auricles. The lungs were free from congestion. The cerebral vessels were fully engorged. In this, as in the former instance, the smell of ether could readily be detected in the brain, on opening the skull twenty-four hours, or more, after the operation.

I have seen the chloroform administered in several instances. In some cases its operation did not differ much from ether, with the exception that insensibility was sooner induced—from one half to two minutes. In one instance, more than the usual quantity failed to procure any thing more than a little vascular excitement. In one case the patient—a young man—was greatly agitated, the face flushed, and when coming out of its first effects he complained of unwonted sounds in the head, ringing in the ears, &c. A young man, who had taken it for the purpose of having a portion of the toe nail excised, observed to me that about half an hour after the operation he was attacked with violent rigors which passed off after some hours. In another case, for a similar operation, a young lady aet. about 20 years, fat and plethoric, took chloroform. It did not produce entire insensibility, but was followed with rigors, a sense of great expansion of the head, and with various spasmodic symptoms during most of the day.

I have never given it to any of my patients, nor do I think I shall, at present. I fear it more than I do ether.

I shall continue my experiments on the lower animals, both with ether and cloroform, and if I notice any results of interest, I will advise you of them. Perhaps the above account will be of no service to any one ; if you so judge, do not clog your pages with it.

